# Modulation effects of microorganisms on tea in fermentation

**DOI:** 10.3389/fnut.2022.931790

**Published:** 2022-08-02

**Authors:** Ting Hu, Shuoshuo Shi, Qin Ma

**Affiliations:** ^1^Key Laboratory for Green Chemical Process of Ministry of Education, Hubei Key Laboratory of Novel Reactor and Green Chemical Technology, School of Environmental Ecology and Biological Engineering, Wuhan Institute of Technology, Wuhan, China; ^2^Key Laboratory of Functional Foods, Ministry of Agriculture and Rural Affairs/Guangdong Key Laboratory of Agricultural Products Processing, Sericultural & Agri-Food Research Institute Guangdong Academy of Agricultural Sciences, Guangzhou, China

**Keywords:** tea beverage, microbial fermentation, tea polyphenols, biological activity, sensory characteristics

## Abstract

Tea is a popular traditional drink and has been reported to exhibit various health-promoting effects because of its abundance of polyphenols. Among all the tea products, fermented tea accounts for the majority of tea consumption worldwide. Microbiota plays an important role in the fermentation of tea, which involves a series of reactions that modify the chemical constituents and thereby affect the flavor and bioactivities of tea. In the present review, the microorganisms involved in fermented tea and tea extracts in the recent studies were summarized and the modulation effects of microorganisms on tea in fermentation, including polyphenols composition and content, biological activities and sensory characteristics, were also critically reviewed. It is expected that the data summarized could provide some references for the development of microbial fermented tea drinks with specific nutrition and health benefits.

## Introduction

Tea, one of the most popular beverages in the world, is generally made from the leaves of the *Camellia sinensis* plant through processing techniques such as curing, rolling, heaping, and drying. Tea contains various bioactive ingredients, such as polyphenols, polysaccharides, caffeine, minerals and other active substances (1). Previous studies have indicated that tea possessed antioxidant, hypoglycemic (2), antihypertensive, lipid-lowering (3), antibacterial, anti-cancer, and anti-obesity effects, which were mainly attributed to its abundant polyphenols (4).

With the rise of fermentation engineering, researchers applied microbes to tea. Unlike green tea, black tea and other teas that have not undergone microbial fermentation, microbial fermented tea has special sensory characteristics, including bright tea infusion, unique aroma, sweet, and smooth taste with low levels of bitterness and astringency. Therefore, dark tea and kombucha, two kinds of microbial fermentation tea, are favored by consumers for their special taste and aroma (5, 6). Furthermore, tea has been fermented by microorganisms that are attracting increasing attention because of its various health benefits, including protection against hypertension and cardiovascular diseases (7, 8). At the same time, the content of its active ingredients will change (9–11). After fermentation, some molecules in tea that are not easy to be absorbed change from combined state to free state, which is conducive to the absorption of nutrients in tea and the exertion of its efficacy. Microbial fermentation is the key factor responsible for the formation of sensory attributes and the chemical components of tea.

The changes of chemical constituents and bioactivities of tea during microbial fermentation have been revealed in recent years, which were related to the bioconversion of active components by microorganisms. The purpose of this paper is to review the types of microbiota, changes of polyphenols content and composition, biological activity and sensory evaluation in tea after microbial fermentation, according to comprehensively understand the characteristics and current situation of microbial fermented tea, and look forward to its development prospects and research trends, which may provide a theoretical basis for the in-depth study of tea fermented by microorganisms.

## Typical types of microbial fermentation in tea

**Figure 1:**
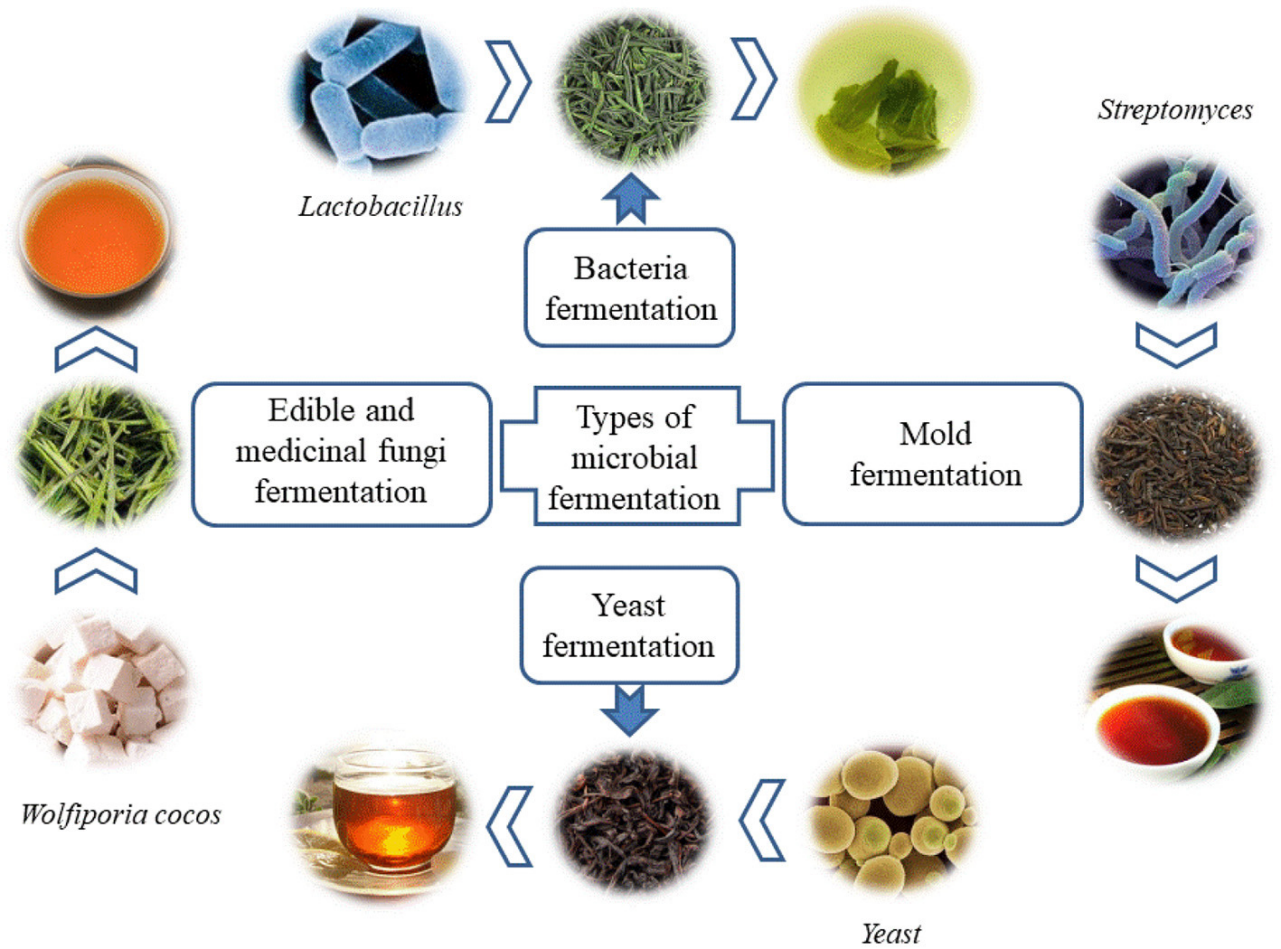
Typical types of microbial fermentation in tea.

According to the type of microbiota, the microbial fermented tea can be divided into four types, including bacterial fermented tea, mold fermented tea, yeast fermented tea and edible and medicinal fungi fermented tea, as shown in Figure 1. After fermentation, the antibacterial activity and antioxidant activity of tea were enhanced due to the increase of phenolic substances. The reduction of caffeine after fermentation makes the tea taste better. In addition, after fermentation, the variety of aromatic substances increases, giving the tea a new aroma. As well as changes in the types and content of tea pigments, giving the tea soup has a clear color. Tea are fermented by microorganisms, a variety of active substances produced by microbial metabolism increased, thereby improving the overall quality of the tea.

### Bacteria fermentation in tea

In recent years, the research on using bacteria to ferment tea is mainly focused on kombucha. During the fermentation process of kombucha, changes in sugar and acid content give the tea a new taste, the production of aromatic substances increases the aroma of the tea, and changes in phenolic substances increase the antioxidant capacity of the tea.

Zhao et al. reported that *lactic acid bacteria* fermentation may be an effective method to improve the bioavailability of phenols and protect cells from oxidative stress (12). Furthermore, kombucha is fermented by *acetic acid bacteria* and yeast, during which the content of beneficial ingredients such as vitamin C and glucuronic acid increases (13). Simultaneously kombucha has significant changes in antioxidant potential, pH, acetic acid, alcohol and sugar contents, and beneficial ingredients such as organic acids, minerals and vitamins, amino acids and polyphenols can be produced in the fermentation process (14).

### Mold fermentation in tea

On account of tea polyphenols in tea have no inhibitory effect on mold, and some metabolites produced by mold will produce a series of reactions in tea, including degradation, oxidation, methylation, etc., which can improve the quality of tea. Therefore, when tea is fermented with mold, some researchers selected *Streptomyces, Aspergillus niger*, and *Mucor* to conduct fermentation studies (15).

The results showed that compared with fresh tea, the polyphenol content of tea fermented by *Streptomyces bacillaris* strain R9 and *Streptomyces cinereus* strain Y11 strains was higher, and the total phenol content was 32.9 ± 0.1 mg/mL and 31.9 ± 0.1 mg/mL, respectively, after 42 days of fermentation (16). In another set of experiments, the contents of polyphenols and purine alkaloids in solid state fermentation system of *Aspergillus niger* and *Aspergillus fumigatu* were experimentally studied, which provided a reliable basis for dynamic data description and metabolic pathway of tea polyphenols in fermented pu-erh tea (17). Similarly, when molds and yeasts were used in the fermentation of black and green teas, molds fermentation increased caffeine content, which are related to the methylation process and the increase of the premise substance of caffeine, while yeasts fermentation decreased caffeine content. It was found that *Aspergillus niger* had the best fermentation performance of the three molds in this study (18).

In addition, *Eurotium cristatum* was also used for tea fermentation in recent years. The bacteria secrete amylase and oxidase, which can catalyze the transformation of protein and starch in tea to monosaccharides, catalyze the oxidation of polyphenol compounds, and transform them into substances beneficial to human body, so as to improve and optimize tea taste and other characteristics. When the extract of raw dark tea was fermented by using *Eurotium cristatum*, the dry weight of mycelia increased by about 10 times after the fermentation, the mass concentrations of tea polyphenols, total protein and water extract were decreased, the concentrations of total flavonoids, free amino acids and theabrownin were increased, and 12 kinds of aromatic components were increased, most of which were esters and alcohols (19).

### Yeast fermentation in tea

Using yeast to ferment tea can not only improve the activity of tea fermentation, but also the complex biochemical reactions in the fermentation process will produce ethanol, acids and esters and other flavor substances to improve the sensory quality of the microbial fermentation of tea.

Studies have shown that the fermentation of black tea by *Dabaryomyces hansenii* results in the reduction of caffeine and a large amount of tannins, and improves its nutritional and medicinal value, so the ingestion of fermented tea is more advantageous than black tea (20). Additionally, when yeast strains were isolated from pu-erh tea and fermented raw tea samples of pu-erh tea, the study found that after yeast fermentation, the contents of tea polyphenols, theaflavins and catechins in tea were increased, while the contents of amino acids, caffeine, flavonoids, thearubigins and theabrownin were decreased. The contents of amino acids, catechins and caffeine affect the taste of tea, and the content of tea pigment determines the color of tea soup. The decrease of theanine content improved the bitter taste of tea, while the increase of theaflavins improved the color, aroma and taste of tea. Therefore, yeast has a great influence on the quality formation of pu-erh tea (21).

### Edible and medicinal fungi fermentation in tea

With the in-depth study of tea and modulation effects of microorganisms on tea beverage in fermentation process, many researchers also introduced edible and medicinal fungi for tea fermentation. With the fermentation of edible and medicinal fungi, the tea fermented by microorganisms goes through biochemical reaction to obtain aroma substances such as esters and alcohols. There were also changes in substances such as polyphenols and proteins. Improved the stale taste, sour taste and astringency of tea, and gave tea a new aroma and taste.

Rigling et al. used *Poria cocos* to adjust the smell of green tea. The study found that after immersion and fermentation for 17 h, due to the formation of methyl anthranilate, linalool, 2-phenylethanol and geraniol, *Poria cocos* changed the unique smell of green tea into jasmine flower and slightly citrus flavor. Meanwhile, the antioxidant activity of green tea is retained (22).

In addition, after the fermentation of Jinxuan oolong tea with medicinal mushrooms *Grifola frondosa* and Tianzhi (new variants of *Ganoderma lucidum*), the contents of polysaccharide, free amino acid and protein of the fermented tea were significantly increased, and the taste of the fermented tea was fresher and mellow. The contents of tea polyphenols, caffeine and water extract in the fermented products were significantly reduced, which reduced the turbidity of tea juice, reduced the bitterness, and gave it sweet taste and aroma (23).

## Changes of polyphenols content in tea after microbial fermentation

Tea polyphenols are the main components that determine the color, aroma, taste and efficacy of tea. They are classified as flavonoid (flavonols, flavanols, flavones, flavanones, isoflavones, and anthocyanins) and non-flavonoid molecules (phenolic acids, hydroxycinnamic acids, lignans, stilbenes, and tannins) (24). Tea polyphenols have anti-inflammatory, antiviral, antibacterial, hypolipidemic, hypoglycemic, weight loss and other effects. After microbial fermentation, the content of polyphenols changes in the tea (25–28).

### Changes of flavonoid content in tea

In recent years, researches on flavonoids polyphenols in tea mainly focus on flavanols, flavonols, and flavones. This section will review the changes of flavonoid content of tea before and after microbial fermentation.

**Figure 2:**
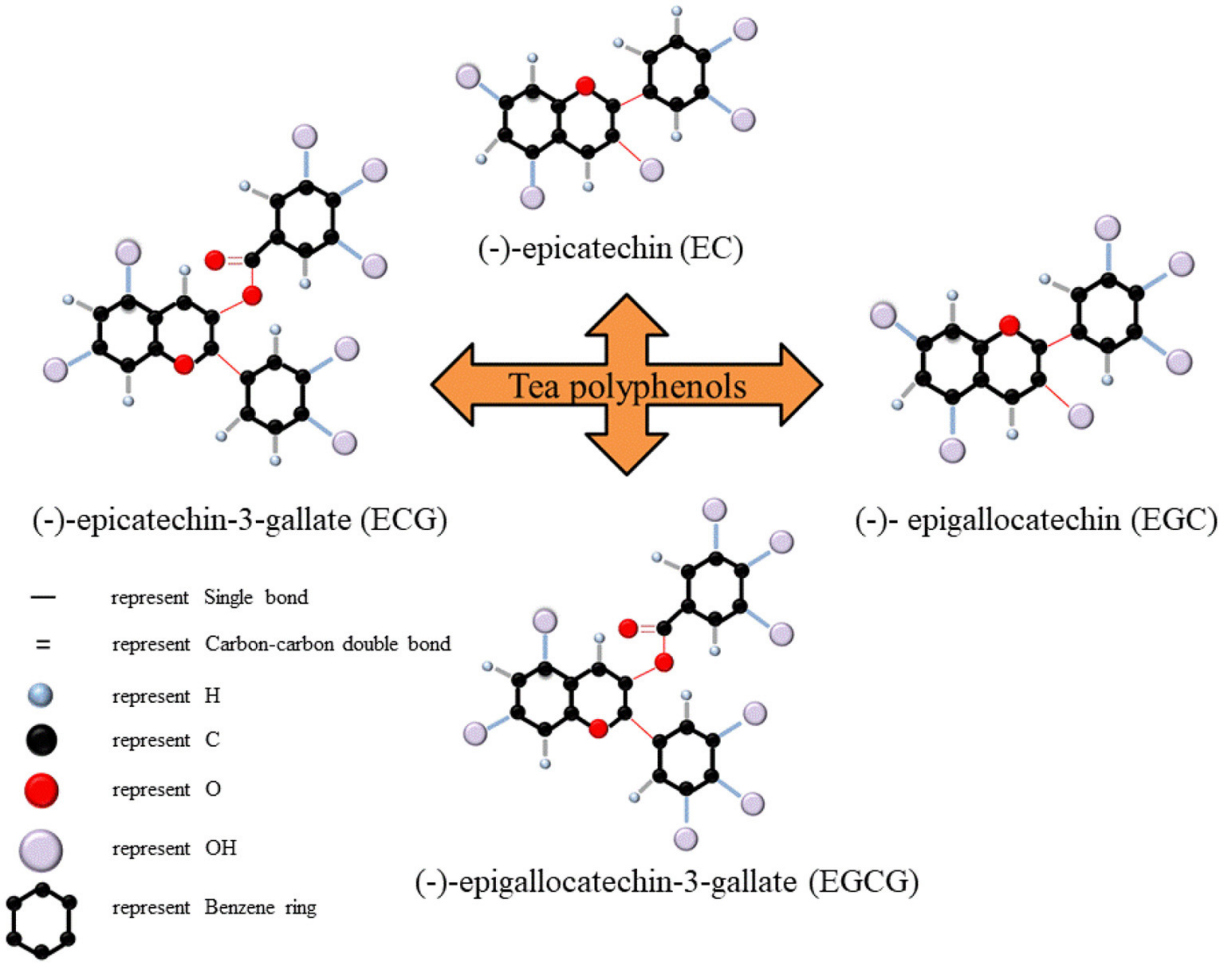
The main structure of tea polyphenols.

Catechins are synthesized from sugars through shikimic acid pathway through the action of a series of enzymes to form benzene ring compounds. Catechins are typical flavanols, accounting for about 18 to 36% of the dry weight of tea (29). As shown in Figure 2, there are four important structures of catechins, namely (-)-epigallocatechin-3-gallate (EGCG), (-)-epicatechin-3-gallate (ECG), (-)-Epicallocatechin (EGC) and (-)-Epicatechin (EC). In the process of tea fermentation, the content of catechin varies with the degree of tea fermentation, fermentation time, fermentation temperature and other factors (30). In another study, Qin et al. using the method of quantitative analysis and high performance liquid chromatography to study the change of tea polyphenols content of pu-erh tea in solid-state fermentation system, it was found that the content of ester-catechins increased slightly at the initial stage of fermentation, and then the ester-catechins gradually degraded to produce catechin and gallic acid. In the initial stage (0 to 8 h), the content of EGCG increased slightly, from 23.392 and 23.431 mg/g to 24.983 and 24.897 mg/g (17). According to another set of study, used the dominant strain of qingzhuan brick tea to conduct solid fermentation of qingzhuan brick tea, and after 6 days of fermentation, the catechin content decreased by 27.6% under the action of *Aspergillus fumigatus* M1 (25). The content of catechins in most teas decreases during fermentation, possibly due to the breakdown of catechins into substances such as theaflavins during fermentation (31).

The main flavonols and flavones in tea include kaempferol, quercetin, myricetin, and apigenin. Wang et al. identified flavonoid glycoside (quercetin-3,4'-O-di-β-glucoside, quercetin 3-O-galactosyl rutin, myricetin 3-galactoside, luteolin 6-C-glucoside, vitexin (apigenin-8-C-glucoside), kathinol 7-O-glucoside) in green tea extracts fermented by *Lactiplantibacillus plantarum* 299 V significantly were decreased, which may be related to the absorption and utilization of flavonoids by *lactic acid bacteria* cell wall (32). Seven kinds of tea fungis were used to ferment the sun-dried green tea, which promoted to the accumulation of kahenol and myricetin. It was found that the antioxidant activity of tea increased after fermentation, and the positive correction of gallic acid and kamanol to the antioxidant activity of fermented tea was observed (33). In addition, Ma et al. found that *Aspergillus palladium* PT-3 and *Aspergillus sesamae* PT-4, two tea fungis, could promote the biosynthesis of various flavonoids such as neferol, quercetin, and myricetin in the metabolic process of phenolic compounds, thus increasing the content of flavonoids in tea during fermentation (34). Furthermore, other researchers have used bacterial strains to ferment black tea to make drinks. For example, when *Starmerella davenportii* strain Do18 was used to fermented black tea extract, the research results showed that the flavonoid content of fermented tea drinks was higher, and the total flavonoid content of fermented drinks was significantly higher than that of unfermented samples, and reached the highest level after 36 h of fermentation (35).

Moreover, changes in acids in the fermentation environment also lead to the release of bound flavonoids (36–38). However, in the study of inoculated fermented tea, the flavonoid content of fermented tea was higher than that of unfermented tea.

### Changes of non-flavonoid molecules content in tea

Phenolic acids in tea are secondary metabolites of aromatic substances and non-flavonoids, which have many biological characteristics. The phenolic acids in tea mainly include gallic acid, chlorogenic acid, salicylic acid, vanillic acid and so on. During the fermentation of tea, the content of phenolic acid will vary with the degree of fermentation (39, 40).

Gallic acid is one of the important active ingredients in tea. Fermentation of tea will affect the content of gallic acid in tea. When studying the fermentation of loose tea by *Eurotium cristatum* at different temperatures, it was found that appropriately increasing the fermentation temperature was beneficial to increase the content of gallic acid in fu brick tea (41).

Liu et al. used *Aspergillus Niger* to ferment tea. It was found that tannase was involved in the metabolism of gallic acid during the fermentation of pu-erh tea, which increased the content of gallic acid during the fermentation (42). However, Gallic acid showed a decreasing trend in the early fermentation stage of dark tea fermented by *M. coronoid* (43). In addition, in the fermentation process of black tea extract, with the increase of fermentation time, it is not conducive to the accumulation of phenolic acids such as gallic acid and cinnamic acid (44). In the fermentation process of tea, the change of phenolic acid content should be analyzed in combination with the specific situation (45).

## Changes of biological activity in tea after microbial fermentation

Tea has biological activities such as antibacterial, antioxidant, hypoglycemic, weight loss, anticancer and so on (46–48). With the fermentation of tea, its active function will change. The antibacterial, anti-oxidant, hypoglycemic, anti-lipid, anti-inflammatory, anti-toxin and anti-cancer activities of microbial fermented tea can be applied to the food, medicine and cosmetics industry and have a good development prospect (49–52). In this section, the antibacterial, antioxidant, hypoglycemic, lipid-lowering and other activities of tea fermented by microorganism would be elaborated.

### Changes in antibacterial activity

**Figure 3:**
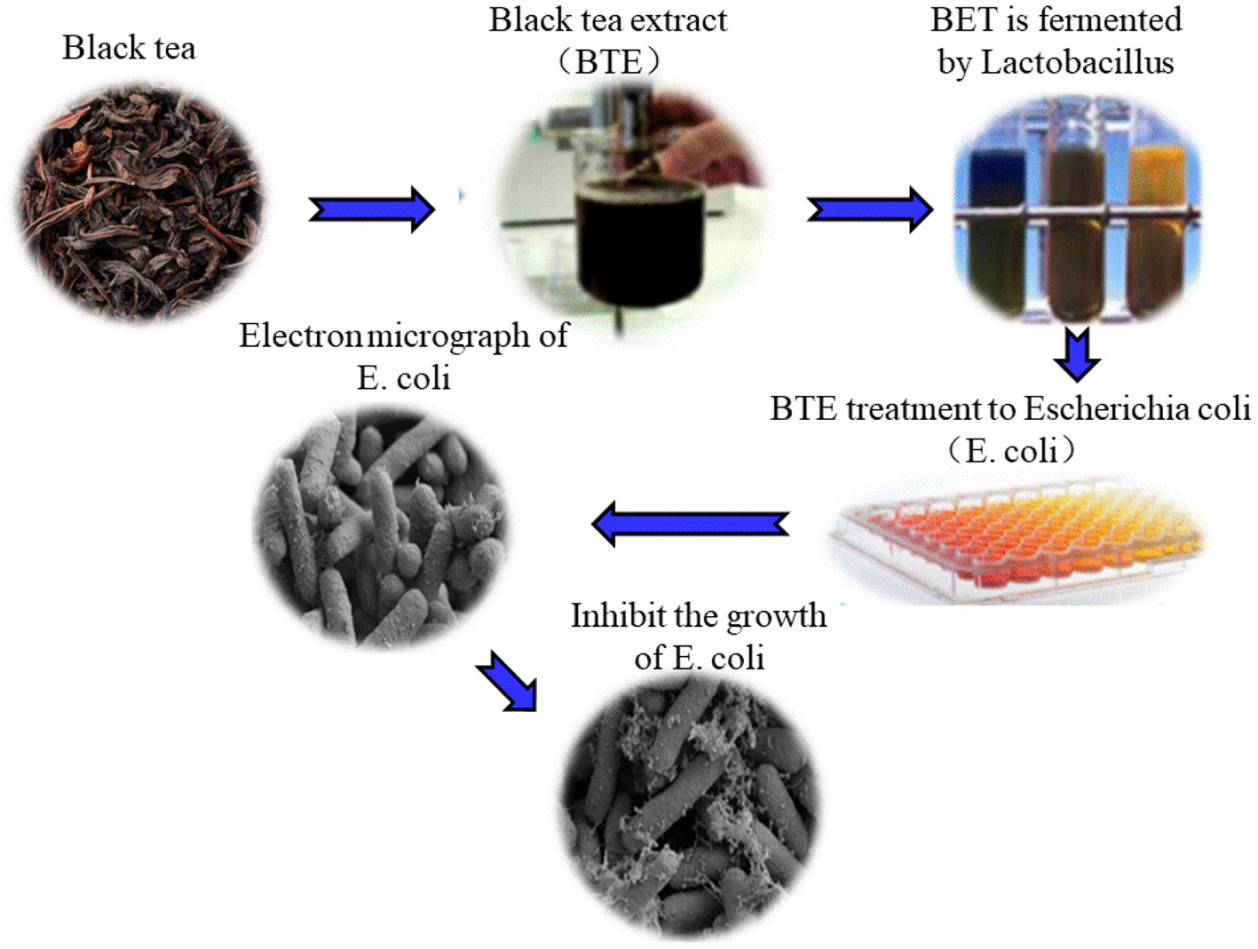
The inhibitory effect of fermented black tea extract on *Escherichia coli*. The cell membrane and cell division of *Escherichia coli* were damaged by 25 mg/ml fermented tea extract.

With the deepening of fermentation, the metabolites of some fungi in tea have the function of inhibiting intestinal microorganisms (53, 54). Comparing the inhibitory effects of black tea extracts before fermentation and black tea extracts after fermentation on *Escherichia coli*, the research results showed that among the three different concentrations of non-fermented black tea extracts, only the tea extract at a concentration of 25 mg/mL can inhibit *Escherichia coli*. The fermented black tea extracts at concentrations of 5, 10, and 25 mg/mL can significantly inhibit the growth of *Escherichia coli*. Figure 3 shows the damage of 25 mg/ml fermented black tea extract to *Escherichia coli* (55). When studying unfermented black tea extracts and fermented black tea extracts, it was found that as the fermentation time increased, the antibacterial effect of black tea extracts increased. The results showed that under the condition of PH 7.0, black tea extracts had no inhibitory effect on *S. typhimurium* when it was not fermented; when fermented for 14 days, the inhibition of *S. typhimurium* by fermented tea could reach 30–35 mm in diameter; Unfermented black tea extracts has no inhibitory effect on *Escherichia coli*, and after 14 days of fermentation, the diameter of the inhibitory zone of fermented tea on *Escherichia coli* can reach 30–35 mm; when fermented for 0–4 days, fermented tea has no inhibitory effect on *Candida albicans*. When fermented for 4–14 days, the inhibition halo diameter of fermented tea on *Candida albicans* reaches 10–15 mm (56). Studies have found that the fermented fu brick tea contains a class of triterpenoids with 6-hydroxy-7-one function. The antimicrobial activity of compound enteric pathogenic *Escherichia coli, Escherichia coli, Staphylococcus aureus, Shigella dysenteriae*, and *Salmonella typhi* was evaluated by plate diffusion. The test results show that the compound has weak antibacterial activity against enteropathogenic *Escherichia coli* (EPEC) and *Salmonella typhi*. The microbial fermented tea has a certain inhibitory effect on harmful bacteria, and the inhibitory ability may be related to the fermentation time, pH and other factors of the microorganisms (57).

In addition, black tea extracts fermented by *acetic acid bacteria* and yeast showed obvious inhibitory effects on *Staphylococcus aureus* ATCC6538 (*S. aureus*) and *E. coli* ATCC11229 (*E. coli*) (58), which suggest that fermented tea might be a potential source of preservatives. The antimicrobial activity of fu-brick tea after fermentation by *bursa corundum* is obviously improved compared with that without fermentation. When the concentration of fermented tea extract was 5 mg/mL or less, the growth of intestinal pathogenic *bacteria Shigella* and *Staphylococcus aureus* could be reduced by 50%, and the minimum inhibitory concentration of fermented tea extract against *Staphylococcus aureus* was 0.625 mg/mL (59). After microbial fermentation, the antibacterial activity of tea was improved, and its antibacterial effect on *Escherichia coli, Staphylococcus aureus, Salmonella* and other pathogenic bacteria had improved significantly.

### Changes in antioxidant activity

During the fermentation of tea, the antioxidant capacity of tea will also change with the changes in content of secondary metabolites (12, 60, 61). A single strain isolated from pu-erh tea was used to inoculate fresh tea to study its fermentation effect. The results of the study showed that fresh tea was inoculated with these strains, and the antioxidant capacity was significantly enhanced after 42 days of fermentation. Among them, the polyphenol content of tea inoculated with *Streptomyces bacillus* strain R9 was 3.3 mg/100 g, and the scavenging ability of DPPH free radicals reached 92%, the total polyphenol content was the highest, and the antioxidant capacity was the strongest (11). Fermentation of fu brick tea using *Eurotium cristatum* has found that fermented tea at 28 and 37°C has strong antioxidant capacity (26). Compared with unfermented tea, fermented tea has better antioxidant properties, and fermentation temperature has a significant impact on the antioxidant properties of fermented tea. Furthermore, using kombucha to ferment black tea, and study the changes in its antioxidant activity with fermentation time, the results of the study found that as the fermentation time increases, the antioxidant activity of fermented tea increases. After 8 days of fermentation, the antioxidant activity of fermented tea can reach up to 89.69% (62). And the DPPH scavenging ability of green tea reached 94.38% after fermentation of GABA-producing *lactic acid bacteria* for 5 days (63).

Combined with the above research, when studying the changes of antioxidant activity of microbial fermented tea, we can improve the antioxidant activity of tea through microbial fermentation, so as to provide a theoretical basis for the research and development of antioxidant functional food and the food industry.

### Changes in hypoglycemic activity

In recent years, many researchers have used animal experiments to study the biological activity of tea and found that after microbial fermentation, it is demonstrated that the activity of tea to reduce blood glucose has been improved (64–66).

**Figure 4:**
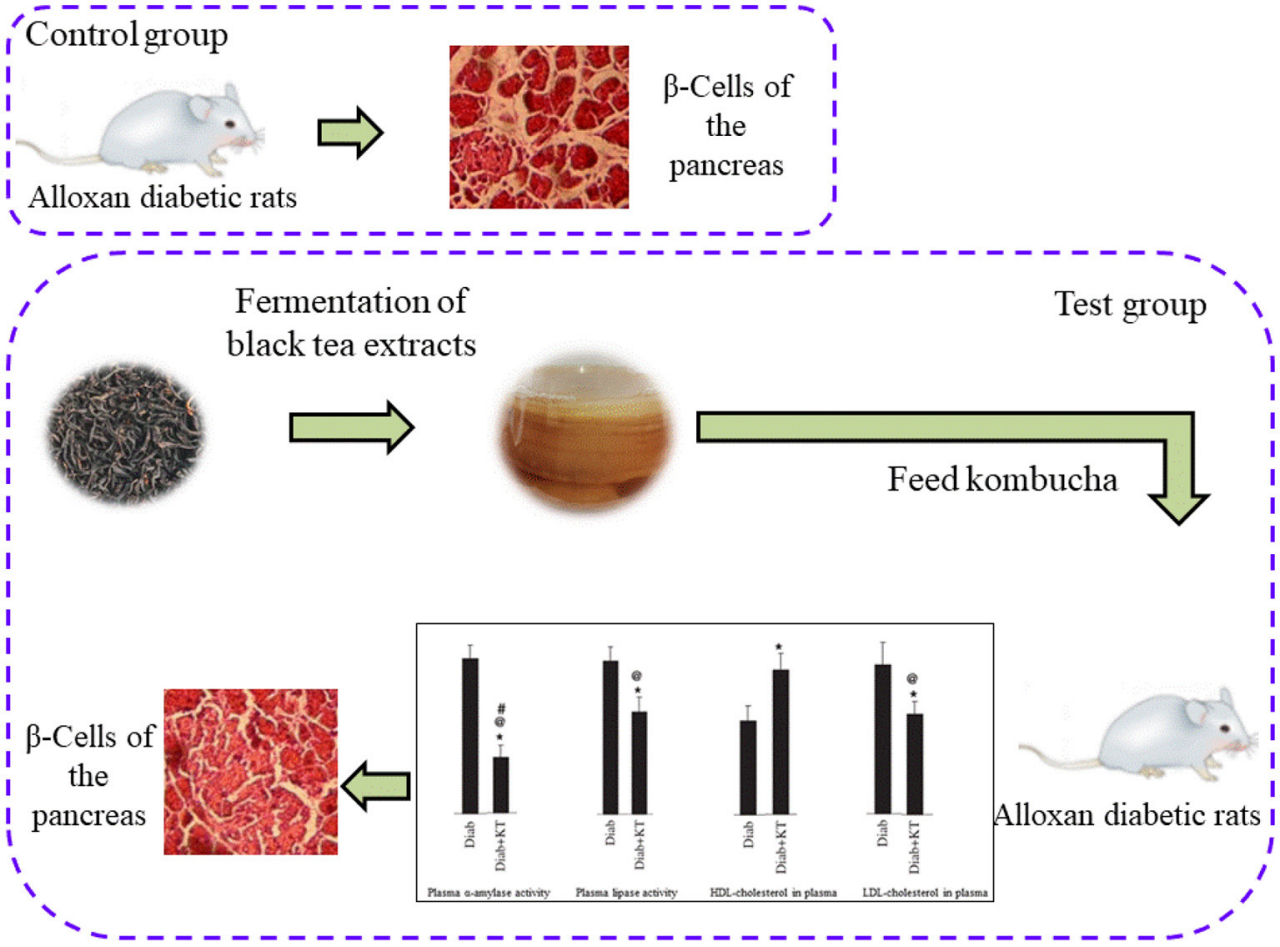
Effect of fermented black tea extract on β-cells of the Pancreas in Alloxan diabetic rats.

The regulation effect of fermented tea on glucose metabolism is mainly related to the metabolites of fermented tea. For example, the relative contents of 10 kinds of polyphenol metabolites (4 kinds of fatty acids, 1 kind of artemisylline derivative, 3 kinds of lysophosphatidylcholine and 2 kinds of triterpenoids) increased while the relative contents of the other 5 kinds of polyphenol metabolites decreased after the fermentation of dark tea by *Cystis canopetiformis*. These metabolites are related to the hypoglycemic activity of fermented tea (67). It was found that the kombucha has obvious therapeutic effect to diabetic rats after fermentation. The findings from the histopathological analyses revealed that those of the alloxan-induced diabetic rats showed clear atrophy of β-Cells. The pancreas of the diabetic rats that were treated with fermented black tea, on the other hand, noted to undergo a marked amelioration. Mainly because after fermentation of tea extract on plasma and alpha amylase and lipase activity in pancreas have better inhibitory effect, at the same time improve the pancreas of diabetic mice structure, have better inhibitory effect on blood sugar levels rise, as shown in Figure 4 (68). The green tea extracts fermented by *acetic acid bacteria, yeast* and *lactic acid bacteria* can improve the intestinal flora of mice. The improvement of intestinal flora reduces the damage of intestinal barrier, thus reducing lipopolysaccharide replacement and inhibiting the occurrence of insulin resistance *in vivo*. In addition, increasing the number of SCFAs-producing bacteria can increase the number of SCFAs, improve the function of islet β cells, and reduce blood glucose by promoting the secretion of gastrointestinal hormones (69).

### Changes in lipid-lowering activity

At present, research on the lipid-lowering activity of microbial fermented tea is mainly focused on lowering blood lipids and weight loss (70, 71). For example, the fu brick tea was fermented by *Eurotium cristatum*, and the water extract of the fermented fu brick tea was fed to high-fat zebrafish. The results showed that when the concentration was 500 μg/mL, the lipid level of the zebrafish was compared with the control group, it decreased by 51.49%. The water extract of fermented *Eurotium cristatum* showed effective lipid-lowering activity on high-fat zebrafish (72). In addition, *Lactobacillus paracasei subsp* was used to fermentation *Houttuynia cordata* leaf tea and green tea, and the anti-obesity activity of fermented tea was studied through in *vivo* and in *vitro* experiments. Wang et al. demonstrated that the fermented tea contained epigallocatechin gallate, epigallocatechin, and chlorogenic acid, which inhibit lipogenesis in mature 3T3-L1 adipocytes by stimulating adipose decompose (73). Qin et al. fermented Anhua black tea with *Monascus* and *Cystaphylococcus coronatum*, resulting in many active substances in the fermented products, such as lovastatin, which had certain lipids lowering effects (74). Therefore, long-term drinking of microbial fermentation tea has a good role in medical care.

### Changes in other biological activities

The tea was fermented by microorganisms has antibacterial, antioxidant, hypoglycemic, lipid-lowering, anti-inflammatory and anti-cancer activities (75, 76). Zhang et al. found that lovastatin produced by monascus fermentation of pu-erh tea can induce neutrophil apoptosis by phosphorylation of ERK/AKT and reduce neutrophil recruitment to inflammatory sites, thereby reducing inflammation in zebrafish (77). In addition, Assam tea after fungal fermentation, compared with unfermented tea, microbial biological conversion of phenol makes total polyphenol, total tannin content in the fermented tea enhancement and enrichment of condensed tannins, thus as a target, the function of the bioactive ingredients in anti-inflammation mediated diseases such as cancer and cardiovascular disease found in the corresponding application (78). Moreover, Villarreal-soto et al. studied the bioactivity of extracts from black tea fermented by kombucha bacteria. After 21 days of fermentation, the anti-inflammatory bioactivity was enhanced, with IC_50_ values up to 9.0 μg/mL (79). Li et al. used *Nigrospora sphaerica* HCH285 to ferment the sun-dried green tea, so as to develop a kind of melanospora fermented tea. With the increase of fermentation time, after 45 days of fermentation, the content of bostrycin in the fermented tea reached 3.18 g/kg, and the content of bostrycin was the highest in the whole fermentation process. Bostrycin has good anticancer activity, so the anticancer activity of green tea is enhanced in the fermentation process (80). Tea are fermented by microorganisms, most of the biological activity of tea will be enhanced.

## Changes of sensory evaluation in tea after microbial fermentation

Not only there is a change in the content of tea polyphenols and bioactive functional, the sensory properties of tea changed after fermentation (81, 82). For example, the change of tea taste and the color and brightness of tea juice are mainly caused by the change of amino acid content in the fermented tea and the formation of theaflavins and thearubigen (83, 84).

### Change in taste

Compared with unfermented tea, the taste of tea is enhanced by microbial fermentation. This is because, after tea fermentation, the protein in tea is decomposed into free amino acids, increasing the flavor of tea. At the same time, catechins were degraded and the bitter taste of tea beverage was reduced. Besides, sugar content increased and pH value decreased, making fermented tea beverage taste sour and sweet.

*Laphet*, a fermented tea from Myanmar, has a bitter taste in the raw tea without fermentation, but the bitter taste of the tea is reduced after fermentation (85). Nishioka et al. used *Lactobacillus* to ferment Awaban tea and found that the free amino acids of fermented tea contained large amounts of theine and glutamate, which enhanced the umami taste of tea (86). At the same time, the quality of Awaban tea is improved by some other ingredients produced by lactic acid bacteria. In addition, Zhao et al. used tea bacteria to discover the extract of pu-erh tea and studied the flavor of tea in the fermentation process. It was found that the total amount of free amino acids increased slightly with the increase of fermentation time, and these free amino acids, as the most important aromatic precursors of fermented tea, could significantly improve the flavor quality of fermented tea. At the same time, due to the presence of *acetobacter*, the PH of fermentation liquid is reduced, tea polyphenols are oxidized, catechins are degraded, and bitterness is reduced, and fermented tea is endowed with sweet and sour taste (87). Moreover, it was found that the sensory properties of black brick tea were changed during fermentation by liquid chromatography-mass spectrometry. Based on liquid chromatography-mass spectrometry of metabonomics analysis revealed that microbial fermentation of the tea samples and microbial fermentation before samples have significant differences, a total of 102 compounds were identified as the key to lead to metabolic changes and green tea processing metabolites, catechins content decreased significantly, and form new phenolic acids and catechins derivatives. The sensory quality of the green brick tea is mainly formed in the process of microbial fermentation, which greatly reduced the astringency and bitterness of raw tea and produced its characteristic woody and stale aroma as well as mellow taste (88). When the dark tea was fermented by *Aspergillus Niger*, the bitterness and astringency of tea were reduced significantly due to the catechin content was decreased in the fermentation process. In addition, bitterness and umami taste were also changed due to amino acids were changed in tea (89). After the fermentation of bacteria, the pH of black tea decreases at the initial stage of fermentation, during which sucrose is hydrolyzed and lactic acid content increases, and tea is endowed with a sour and sweet taste (5).

### Change in aroma

Tea has been fermented by microorganisms. Aroma substances in tea are produced, including alcohols and aldehydes. Besides enzymatic and non-enzymatic reactions occur in tea, and the hydrolysis of some substances, such as glycoside hydrolysis, has a great influence on the formation of tea aroma (90–92).

For example, in the post-fermentation process of dark tea, ketones are formed. These ketones are very important flavor compounds with special floral and woody odors. Therefore, the fermented dark tea has a special fragrance (93). In the fermentation process of pu-erh tea extract, the aroma of tea becomes weaker under the action of microorganisms, while the compositions of fermented tea such as camalool, phenylacetaldehyde, heptanaldehyde and 2, 4-dimethylbenzaldehyde are produced, which makes the tea fermentation liquid have the fruit flavor (60). In addition, the study used four non-Saccharomyces cerevisiae strains to ferment green tea slurry, and the proper fermentation of these four strains changed the characteristics of aroma compounds, so that the aroma of fermented green tea changed. In the microbial fermentation process of tea, the aroma composition and aroma will change benignly, which makes the sensory sense of tea more abundant (94). After the fermentation of green tea with four kinds of mixed bacteria, a fruity ethyl ester was produced, which increases the content of aroma compounds such as methyl salicylate, geraniol and 2-phenylethanol, giving tea a special aroma (32). Furthermore, the presence of D-limonene (27.71%) and β-cinnamene (11.55%) in fu brick tea was fermented by *Eurotium cristatum* gives the tea a fruity and special aroma compared with the unfermented green tea (72). Besides, *Cyberlindnera aturnus var. mrakii* NCYC 2251 was used to ferment green tea pulp. With the extension of fermentation time, glycosylated aroma substances such as methyl salicylate, benzyl alcohol and 2-phenylethanol increased (95).

### Change in color

After the tea was fermented, the taste and aroma would be changed. What's more, the color of tea will also be changed. Under the action of microbial fermentation, the content and type of tea pigment in tea change, and then change the color of tea soup. Therefore, the color of tea beverage will become bright, and the tea soup will be improved after microbial fermentation.

**Figure 5:**
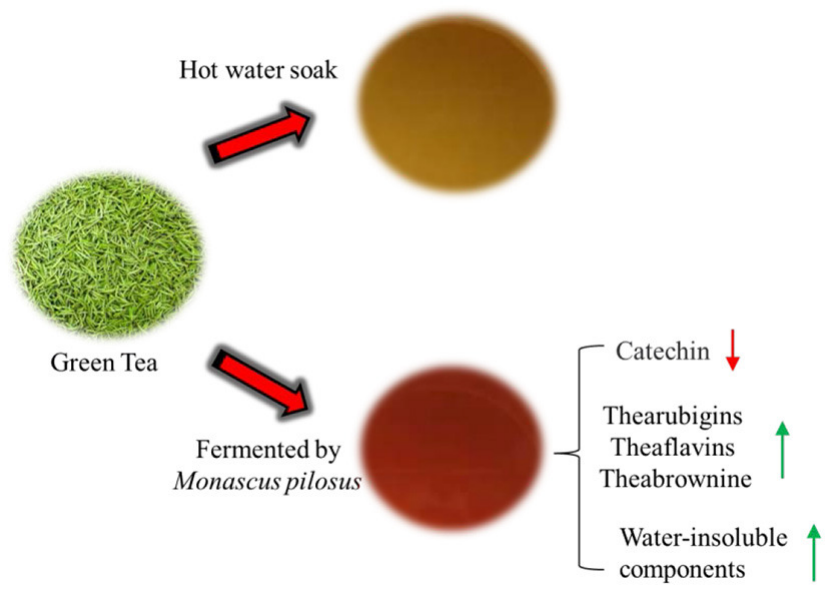
The color change of tea soup before and after fermentation of Green Tea.

For example, the brown pigment content affects the color of black tea. After fermentation with *Aspergillus fumigata M1*, the brown pigment content of pu-erh tea extracts increases by 110.6%, which improves the color of pu-erh tea (25). Kim et al. conducted sensory evaluation on *Monascus Pilosus* fermented green tea and found that the brightness evaluation of fermented tea soup was as high as 4.26, significantly higher than that of unfermented tea. Therefore, microbial fermentation of tea can improve the color of tea soup and the overall quality of tea (96), as shown in Figure 5. After fermentation, the color of the tea soup mostly become translucent and the overall quality will be improved.

## Concluding remarks

In the process of microbial fermentation of tea, the content of catechins and flavonoids in tea mostly showed a decreasing trend, mainly due to the oxidation or degradation of catechins and flavonoids into gallic acid during the fermentation process. The biological activity of unfermented tea and microbial fermented tea is also different. After the tea is fermented, its antibacterial and antioxidant capacity would be enhanced. In addition, microbial fermented tea also has anti-inflammatory and weight loss effects. Long-term drinking of microbial fermented tea has good medical and health care effects. Compared with unfermented tea, microbial fermented tea has improved sensory performance, reduced bitterness and astringency, stronger aroma, and brighter tea juice. At present, researchers have not done much research on the fermentation of tea with inoculated microorganisms. In the future, the fermentation of tea with inoculated microorganisms may have a good development prospect. At present, there are many researches on fermented tea in the field of food, such as kombucha, which uses mixed strains to ferment different kinds of tea. Tea fermented by microorganisms has a good prospect in functional food research and development because of its antioxidant, hypoglycemic and lipid lowering properties. The antibacterial properties of fermented tea have a good application prospect in food preservation. In recent years, with the development of fermentation engineering, bostrycin and lovastatin obtained by microbial fermentation of tea have anti-cancer and anti-inflammatory effects, and are expected to be applied in medical clinical research. This article will provide a theoretical basis for future researchers to explore more the functional activities of microbial fermented tea, and provide a certain scientific basis for the research and development of tea in the field of functional health products, food and medicine.

## Author contributions

TH: conceptualization, project administration, writing—original draft, and writing—review and editing. SS: investigation and writing—original draft. QM: conceptualization and writing—review and editing. All authors contributed to the article and approved the submitted version.

## Funding

This research was financially supported by the National Natural Science Foundation of China (21702156), Hubei Provincial Natural Science Foundation of China (2017CFB200), Graduate Innovative Fund of Wuhan Institute of Technology (CX2021452), and the Special fund for scientific innovation strategy-construction of high level Academy of Agriculture Science (R2019YJ-YB1003).

## Conflict of interest

The authors declare that the research was conducted in the absence of any commercial or financial relationships that could be construed as a potential conflict of interest.

## Publisher's note

All claims expressed in this article are solely those of the authors and do not necessarily represent those of their affiliated organizations, or those of the publisher, the editors and the reviewers. Any product that may be evaluated in this article, or claim that may be made by its manufacturer, is not guaranteed or endorsed by the publisher.
